# Chemical Composition and Mechanical Properties of Wood after Thermal Modification in Closed Process under Pressure in Nitrogen

**DOI:** 10.3390/ma17071468

**Published:** 2024-03-22

**Authors:** Juris Grinins, Guntis Sosins, Prans Brazdausks, Janis Zicans

**Affiliations:** 1 Latvian State Institute of Wood Chemistry, 27 Dzerbenes Street, LV-1006 Riga, Latvia; guntis.sosins@kki.lv (G.S.); prans.brazdausks@kki.lv (P.B.); 2 Institute of Polymer Materials, Faculty of Materials Science and Applied Chemistry, Riga Technical University, 3/7 Paula Valdena Street, LV-1048 Riga, Latvia; janis.zicans@rtu.lv

**Keywords:** birch, pine, thermal modification, nitrogen, pressure, chemical composition, mechanical strength

## Abstract

In this study, silver birch (*Betula pendula*) and Scots pine (*Pinus sylvestris*) wood planks (1000 × 100 × 25 mm) were thermally modified in pilot-scale equipment. Research extended our knowledge of the thermal modification (TM) process in a closed system under nitrogen pressure, as well as how process parameters affect the chemical composition and mechanical strength of wood. Various TM regimes were selected—maximum temperature (150–180 °C), modification time (30–180 min), and initial nitrogen pressure (3–6 bar). Chemical analyses were performed to assess the amount of extractives, lignin, polysaccharides and acetyl group content following the TM process. The mechanical properties of TM wood were characterized using the modulus of rupture (MOR), modulus of elasticity (MOE), and Brinell hardness. The MOR of both studied wood species following TM in nitrogen was reduced, but MOE changes were insignificant. The Brinell hardness of TM birch wood’s tangential surface was much higher than that of the radial surface, although Scots pine wood showed the opposite pattern. TM birch and pine wood specimens with the highest mass loss, acetone soluble extractive amount, and the lowest xylan and acetyl group content had the lowest MOR and Brinell hardness.

## 1. Introduction

Wood is a natural, sustainable, aesthetically beautiful, and renewable material that has been used for thousands of years in various applications, most notably furniture and building construction. Wood offers numerous advantages, including a beautiful appearance, great strength, low density, and excellent insulating properties. However, wood has numerous disadvantages, including excessive moisture and water uptake, poor dimensional stability, low biological durability, poor fire resistance, a soft surface, and poor weathering stability. As a result, wood is an excellent raw material for a variety of modifications. Any wood that has been chemically, physically, or thermally treated to improve its qualities is commonly referred to as modified wood. While increasing decay resistance is the goal of many wood modification techniques, adjustments can also improve dimensional stability while reducing hydrophilicity.

Thermal modification (TM) processes are the most chemical-free, technologically straightforward treatments available. These procedures involve the TM of wood in a variety of alteration conditions, including steam, air, nitrogen, vacuum, and vegetable oils [1]. TM, as opposed to chemical modification (acetylation, furfurylation, impregnation with thermosetting resins, etc.) or biocide protection, is a simple technique to improve the dimensional stability and biological durability of wood. Impregnation and chemical modification methods necessitate the use of chemicals, as well as the drying and curing of treated wood, which demands additional energy and time. TM improves wood properties, but it affects strength and flexibility. Changes in wood characteristics following TM are linked to changes in its chemical and anatomical structure. Several wood TM processes are available in Europe, with varying pressure and oxygen exclusion. The most common commercialized processes are Thermowood (Finland), PLATO-wood (Netherlands), Le bois Perdure and Rétification process (France), Wood treatment technology (WTT, Denmark), Termovuoto (Italy), Firmolin (The Netherlands), Opel-Therm-Vacuum (Germany), Feuchte–Wärme–Druck (Germany), and Oil-heat treatment (Germany) [2,3,4]. Our earlier study described the precise conditions and discussions of these processes [5].

TM of various wood species in a nitrogen atmosphere at atmospheric pressure has been studied extensively in the scientific journals. But since the 1970s and 1980s, nothing has been reported about the closed TM process under nitrogen pressure and the characteristics of the TM wood [6,7]. In small-scale laboratory reactors, TM in a nitrogen environment was typically carried out for 1 to 8 h, with a total treatment time of from 20 to 50 h, over a broad maximum temperature range of 130 to 260 °C in a variety of studies [8,9,10,11,12,13,14].

Ash (*Fraximus excelsior* L.) wood was thermally modified in a nitrogen flow for 2, 6, and 10 h at 190 °C. The nitrogen flow rate remained constant throughout the TM process, including the cooling stage. It was noted that TM of ash wood at 190 °C in a nitrogen atmosphere for 10 h induced a considerable decrease in the xylose content (from 20.8% to 8.0%) but only a modest decrease in the glucose content (from 58.8% down to 54.9%) [15].

Black poplar (*Populus nigra* L.) wood was thermally modified in a nitrogen environment at temperatures of 160, 170, 180, 190, 200, and 220 °C for 2, 4, 6, and 8 h, for a total process time of 168 h. With the exception of lignin, the TM temperature significantly affected the content of structural compounds, whereas the TM time only significantly influenced the content of chloroform-ethanol extractives. The hemicellulose concentration after TM ranged between 3% (at 220 °C) and 26% (at 160 °C), which was lower than the 30% found in non-modified black poplar wood. The chloroform-ethanol extractives and cellulose content were increased from 1.8% to 6.6% and 52.2% to 61.6%, respectively. The holocellulose content decreased from 82.1% to 64.8%; however, the lignin content change was minor [16,17].

Sapwood of Acacia hybrid (*Acacia mangium × auriculiformis*) was heat-treated in a nitrogen oven under laboratory conditions for 2–6 h at 210–230 °C. Surface examination revealed that more lignin and extractives were present on the wood surface, whereas the concentration of hydroxyl groups and hemicelluloses was reduced. The Oxygen/Carbon (O/C) ratio declined linearly with mass loss, indicating that the content of lignin and extractives (or their degradation products) on the heat-treated wood surface was increased [10]. Larch (*Larix gmelinii*) and red oak (*Quercus rubra*) were thermally modified in nitrogen environments at 200, 250, 300, and 400 °C. The O/C ratio fell gradually during TM. The untreated control O/C ratio was 0.89 for larch wood and 0.97 for red oak wood; however, after TM, these values were reduced to 0.50 and 0.26, respectively [18].

Teak (*Tectona grandis* L.f.) was thermally modified at 220 °C for 20 h in a nitrogen environment. After heat treatment, the holocellulose and hemicellulose contents of short rotation teak (SRT) and long rotation teak (LRT) decreased. As a result of hemicellulose destruction, the relative lignin content increased. The overall extractive content of SRT and LRT decreased due to the evaporation of volatile components [8]. TM of birch (*Betula* spp.), aspen (*Populus tremula*), and grey alder (*Alnus incana*) in saturated steam under pressure at 160 °C for 3 h and 170 °C for 1 h enhanced the content of acetone-soluble extractives by a factor of 2–7 compared to untreated wood. The major portion of natural wood extractives decomposed during heat treatment, and the increase in soluble extractives was caused by hemicellulose breakdown. The amount of extractives decreased as the maximal modification temperature increased, resulting in the creation of easily volatile compounds that were obtained afterwards using extraction [19].

To prevent warping, *Mimosa scabrella* and *Pinus oocarpa* wood samples were fastened between iron supports before being placed in an oven and heat-treated in a nitrogen environment at 180, 200, and 220 °C for 1 h. Before conducting the experiment, the air was removed to create a vacuum, and nitrogen was added at a concentration of 0.6 kgf cm^−2^. Increasing the TM temperature caused greater alterations in the chemical components. Holocellulose was the primary wood chemical component destroyed by heat treatment, with reductions of 65.9 and 73.0 to 60.3 and 70.3% for *P. oocarpa* and *M. scabrella* wood at 220 °C, respectively. Following TM at 220 °C, *M. scabrella’s* lignin concentration grew from 32.3% to 36.8%, while *P.oocarpa’s* increased from 25.7% to 26.9%. At 220 °C, the total extractive content of *P. oocarpa* and *M. scabrella* woods increased from 1.8% to 3.9% and 1.4% to 2.9%, respectively [11].

According to some research, the mechanical strength of wood following TM in nitrogen is diminished, but it can also improve. A 1.8 m^3^ pilot-scale reactor was used to investigate the dimensional stabilization of wood using the *Feuchte-Wärme-Druck* procedure at 180–200 °C and nitrogen environment of 8–10 bar. After 1.5, 2.5, and 3.5 h of TM at 185 °C in a nitrogen flow at 10 bar pressure, the modulus of rupture (MOR) and modulus of elasticity (MOE) of beech wood (with initial densities of 880 and 700 kg×m^−3^) were lowered. Birch wood (initial density: 560 kg×m^−3^) MOR was lowered, but MOE improved. Poplar wood (initial density: 410 kg×m^−3^) and pine wood MOR and MOE were reduced following TM in nitrogen at 195 °C for 2.5 and 185 °C for 3 h, respectively [6].

Radiata pine (*Pinus radiata* D.), Scots pine (*Pinus sylvestris* L.), and Norway spruce (*Picea abies Karst*) boards were treated with an industrially utilized two-stage heat treatment process at temperatures ranging from 165 to 185 °C for 0, 30, 45, 60, or 90 min. MOR of all investigated species after TM was reduced, whereas MOE increased. After TM, the Brinell hardness parallel to the grain increased significantly, but the hardness perpendicular to the grain only marginally increased [20]. Black poplar (*Populus nigra* L.) wood MOR decreased after TM in a nitrogen environment at temperatures ranging from 160 to 220 °C for 2 to 8 h. MOR decreased with increasing modification temperature and time. The highest decrease in MOR, at approximately 44%, was found following TM at 220 °C for 8 h. Only higher temperatures and longer modification times (at 220 °C for 2 h, 4 h, 6 h, 8 h and at 200 °C for 6 h and 8 h) resulted in a modest decrease in MOE. The remaining versions had higher or similar MOE values [17].

TM treatment in a dynamic nitrogen environment at temperatures of 200, 220, and 240 °C increased the resistance of pine (*Pinus taeda*) and *Corymbia citriodora* wood to white and brown rot fungi. TM at 160 and 180 °C increased the biological resistance of *C. citriodora* wood, but showed little benefit for *Pinus taeda* [12]. The European species black poplar (*Populus nigra* L.), European beech (*Fagus sylvatica* L.), European ash (*Fraxinus excelsior* L.), European oak (*Quercus robur* L.) and Scots pine (*Pinus sylvestris* L.) were thermally modified in a nitrogen environment at 190 °C for 6 h. Beech and oak woods’ Brinell hardness decreased following TM, although the variations in poplar, ash, and pine wood were not statistically significant [21].

In this work, we examined the TM of silver birch (*Betula pendula*) and Scots pine (*Pinus sylvestris*) wood, focusing on chemical composition and mechanical strength resulting from treatment. This research is related to our prior study, which evaluated the water-related properties of the same wood species [5]. Birch and pine wood were chosen as the most common deciduous and coniferous wood species in the Republic of Latvia due to their numerous applications in the national economy. Our study aimed to evaluate the relationships between TM parameters in an isolated nitrogen atmosphere under pressure, changes in TM wood chemical components, and the impact on material mechanical strength. The existing information on the nitrogen TM process parameters, particularly the initial pressure, is not shown or discussed. Therefore, conducting a thorough analysis of this TM method makes sense as the next step for validating, adding to, or revising available information.

## 2. Materials and Methods

### 2.1. Materials

Silver birch (*Betula pendula*) and Scots pine (*Pinus sylvestris*) wood boards were purchased from local wood manufacturing companies Selko ltd and Mars ltd, respectively. Each TM used 20 silver birch and Scots pine wood boards measuring 1000 × 100 × 25 mm (longitudinal × tangential × radial). The boards used were of the highest quality, with no visible material flaws (knots, grain slop, resin pocked, bark pocked, reaction wood, wanes, blue stains, decays, bug holes, shakes, distortions, etc.). Before TM, all boards were conditioned in a typical climate (20 ± 2 °C, 65 ± 5% relative humidity). Sapwood and heartwood were not removed from Scots pine wood, and randomly selected boards with specific density limits were used for TM. ISO 13061-1:2014 [22] was used to assess the moisture content of the wood, and ISO 13061-2:2014 [23] was used to calculate the density.

### 2.2. Thermal Modification Process

TM was carried out in a pilot-scale chamber made of grade 304L stainless steel and built by Wood Treatment Technology (Grinsted, Denmark). Hot mineral oil was circulated to maintain a stable temperature inside the jacket. The modification chamber can function at pressures between 0.1 and 20 bar and temperatures up to 190 °C. The equipment is automatically controlled by a program, which enables it to alternate between manual and automatic modes during the TM process. The samples were kept in the autoclave for 30 min at 0.2 bar vacuum to eliminate oxygen before being heated. Following the vacuum step, nitrogen was pumped into the autoclave from a nitrogen gas cylinder to produce the necessary starting pressure (3–6 bar). Initially, a small amount of water (1–1.5 L) was put into the autoclave to generate a small amount of steam to catalyse the hydrolysis of hemicelluloses. The TM system remained sealed and static until the pressure release step (no mixing occurred). Table 1 displays the TM parameters for silver birch that were chosen and tested in our earlier study [5], excluding the additional regime B/150/120/5. T_max_, time at T_max_, and initial nitrogen pressure ranged from 150 to 170 °C, 30 to 120 min, and 3 to 6 bar, respectively.

Scots pine TM parameters: T_max_, time at T_max_, and initial nitrogen pressure ranged from 160 to 180 °C, 30 to 180 min, and 4 to 6 bar, respectively (Table 2). The investigated TM parameters were also chosen and tested in our earlier study [5], except regime P/160/60/5.

**Figure 1:**
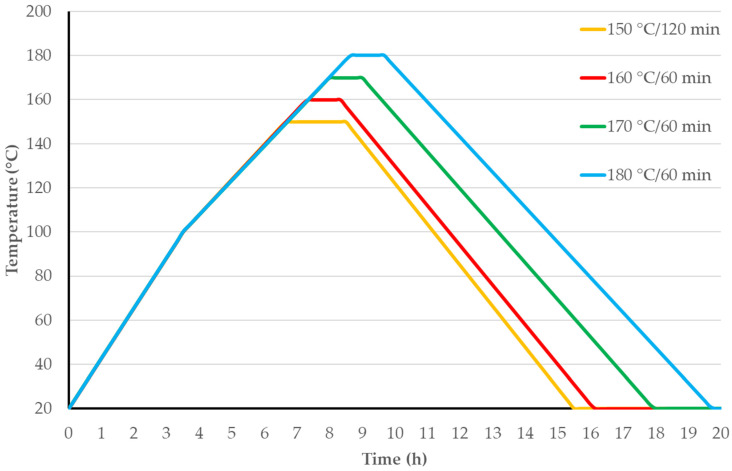
TM regime diagrams at various T_max_.

The modification chamber heated at a rate of 0.36–0.42 °C/min from room temperature to 100 °C, and then at 0.24–0.32 °C/min from 100 °C to T_max_. The temperature was maintained after heating (30–180 min). The pressure in the autoclave increased throughout the TM process, peaking at the T_max_ stage. After that, a mixture of nitrogen and wood thermal destruction products was pumped out of the chamber until atmospheric pressure was attained. To provide further cooling, the modification chamber door was partially opened, and the cooling rate was 0.30–0.35 °C/min. An illustration of the theoretical TM diagrams used in this study is provided in Figure 1. T_max_ was attained in 7–10 h. Depending on the maximum TM temperature, the total duration of the TM process, which included heating, maintaining temperature, and cooling, varied from 16 to 19 h.

### 2.3. Physical Parameters

The mass loss (ML) was estimated by weighing wood boards (20 for each treatment) before and after the TM in a nitrogen atmosphere. After each TM, the ML was determined as a percentage of the original mass of the thoroughly dried wood using Equation (1):

(1) $$ML(\%)=\frac{\left(m_{0}-m_{TM}\right)}{m_{0}}\times 100$$

where:

- ML is the mass loss after TM in nitrogen [%];
- $m_{0}$ is the oven-dried mass of the boards before treatment [g];
- $m_{TM}$ is the oven-dried mass of the boards after TM in nitrogen [g].

### 2.4. Chemical Analyses

Air dried wood sawdust (fraction less than 0.50 mm) from each specimen (32–42 g) was extracted with acetone (99.8%, Acros organics, Geel, Belgium) in a Soxhlet apparatus for 8–10 h. The extractive content was determined for each treatment using Equation (2):

(2) $$EXT(\%)=\frac{\left(m_{0}-m_{E}\right)}{m_{0}}\times 100$$

where:

- EXT is the extractives content [%];
- $m_{0}$ is the oven-dried mass of the specimen before extraction [g];
- $m_{E}$ is the oven-dried mass of the specimen after extraction [g].

After the separation of extractives, structural carbohydrates (glucose, xylose, galactose, arabinose, mannose), acetyl groups (recalculated from the determined acetic acid) and lignin (acid-soluble and acid-insoluble) were determined in the obtained solid residue. The structural carbohydrates were isolated by the two-step sulfuric acid hydrolysis method, according to the National Renewable Energy Laboratory (NREL) standard TP-510-42618 [24]. In the first stage, biomass was treated with 72% *v*/*v* for 60 min at 30 °C in the pressure tube. Periodically, the mixture was mixed with a Teflon rod. In the second stage, deionized water was added to the mixture in such an amount that the concentration of H_2_SO_4_ was 4%. The pressure tube was closed and autoclaved for 60 min at 121 °C. Acid-insoluble residue was removed from the hydrolysate using a filtering crucible with pore size < 8 μm. The concentrations of monosaccharides in the hydrolysate were determined by the Shimadzu LC20AD high-performance liquid chromatograph (HPLC) equipped with an RI detector (Shimadzu RID 10A, Shimadzu, Kyoto, Japan) and a Thermo Scientific HyperREZ XP Carbohydrates Pb^2^⁺ column (Thermo Fisher Scientific, Waltham, MA, USA). The analysis was performed at an oven temperature of 70 °C, employing Milli-Q water as the mobile phase with a flow rate of 0.6 mL/min. Analysis time was 35 min. Prior to HPLC analyses, neutralization of sulfuric acid was achieved using barium carbonate (99–101%). The HPLC method was also employed to analyse by-products of the hydrolysis process, including formic acid, acetic acid, levulinic acid, 5-hydroxymethylfurfural, and furfural, without neutralizing the hydrolysate. For this purpose, the Shodex Sugar SH-1821 column was used at 50 °C, with 0.005 M H_2_SO_4_ as the eluent and a flow rate of 0.6 mL/min, resulting in an analysis time of 55 min. Analytical standards, encompassing cellobiose (≥99%), D-(+)-glucose (≥99%), D-(+)-xylose (≥99%), D-(+)-galactose (≥99%), L-(+)-arabinose (≥99%), D-(+)-mannose (≥99%), formic acid (≥95%), acetic acid (≥99%), levulinic acid (≥98%), 5-hydroxymethylfurfural (≥99%), furfural (≥99%), and sulfuric acid (95–97%), were purchased from Merck (Darmstadt, Germany) and utilized without further purification. The determination of acid-insoluble residue (Klason lignin) was carried out following the NREL TP-510-42618 standard [24], while acid-soluble lignin was determined using the UV-spectrometer Perkin Elmer lambda 650 (PerkinElmer, Waltham, MA, USA) at a wavelength of 203 nm. Additionally, the ash content in the extractive-free wood samples was determined following the NREL TP-510-42622 standard [25].

### 2.5. Mechanical Strength Tests

Before proceeding through mechanical strength testing, the specimens were conditioned in a typical climate (20 ± 2 °C, 65 ± 5% relative humidity). The modulus of rupture (MOR) and modulus of elasticity (MOE) tests were carried out in accordance with ISO 13061-3:2014 [26] and ISO 13061-4:2014 [27]. The Zwick Roell Z010 material testing device (Ulm, Germany) was used to evaluate the static bending strength (3-point flexure test) of 30 specimens measuring 360 × 20 × 20 mm^3^. The Brinell hardness of 10 specimens for each treatment with dimensions of 360 × 50 × 20 mm^3^ (L × T × R) was determined according to EN 1534:2000 [28] using the material testing device Zwick Roell Z100 (Ulm, Germany). Each specimen had 5 indentations made on its tangential and radial surfaces using a hardened steel ball with a diameter of 10 ± 0.01 mm. After removing the indenter for at least 3 min, the measurement rig diameters along and across the grain were measured with an accuracy of ±0.2 mm. The average value was used to determine Brinell hardness.

## 3. Results

### 3.1. Chemical Composition

As a result of TM, silver birch wood underwent ML due to the breakdown of structural wood components, namely xylan and acetyl groups, resulting in increased extractive content (Table 3). For birch wood, the ML following TM varied from 5.9 to 12%. After TM at regimes B/160/120/4 and B/170/60/4, birch wood had the greatest average ML (10.1–12.0%). Following TM at 160 °C for 60–90 min, the lowest average ML was recorded (regime B/160/60/4, B/160/90/3, and B/160/90/4). A higher ML after TM was obtained by raising initial pressure, provided that all other process parameters stayed the same. With the exception of regime B/160/90/4, which displayed statistically significant ML differences from B/160/120/4 and B/170/60/4, the ML values reported by TM displayed broad error margins. The inorganic part (ashes) of untreated and all TM wood specimens was 0.2–0.3%; hence, the data are not given. The most thermally unstable wood cell wall polymers, hemicelluloses, are amorphous polysaccharides that break down to produce most of the ML. Their breakdown frequently starts with the cleavage of acetyl groups from hemicellulose (xylan) side groups, which produces acetic acid and catalyses further breakdown [29]. Our earlier research found that TM promotes shrinkage in the tangential, radial, and total volume of birch wood. The reduction in the tangential direction of TM birch wood was higher (4.4–6.4%) than in the radial (2.9–4.7%), leading to volumetric reductions in the whole board ranging from 7.1 to 10.2% [5].

The chemical components in silver birch wood changed due to TM in a nitrogen atmosphere. The most significant alterations were seen in the content of acetone extractives, xylan, and acetyl groups, while glucan and lignin showed less significance. The percentage of acetone-soluble extractives in TM wood increased 2–6 times (4.0–12.6%) when compared to untreated birch wood (1.9%). A similar pattern was seen for birch (*Betula* spp.), aspen (*Populus tremula*), and grey alder (*Alnus incana*) following TM in saturated steam under pressure at 160 °C for 3 h and 170 °C for 1 h. It resulted in increased content of acetone-soluble extractives by a ratio of 2–7 [19]. In most research studies, TM of wood resulted in higher levels of extractives. However, the opposite impact has also been seen [8]. Surprisingly, the maximum amount of extractives (12.6%) was recovered from wood TM in regime B/160/120/4, while regime B/170/60/4 produced 9.6%. As a result, the most extractives were recovered from the samples with the highest ML after TM. The extractive content climbed as the T_max_ time increased. The initial pressure increase exhibited no apparent trend.

The lignin content increased significantly after treatments B/150/120/5, B/160/60/4, B/170/30/4, and B/170/60/4, but declined for the remainder. The relative content of lignin remained similar regardless of the TM parameters utilized. Because cellulose degrades exclusively in amorphous regions, TM wood contains a greater proportion of crystalline cellulose. The preferential breakdown of amorphous polysaccharides leads to a proportionate rise in lignin content. Despite increased lignin concentration, it still experiences chemical changes during thermal modification, such as de-polymerization and re-polymerization [30]. Furfural and hydroxymethylfurfural are examples of hemicellulose breakdown products that can react with lignin to enhance the concentration of lignin [31].

The relative amount of glucan after TM in nitrogen increased by 0.6 to 4.4%, with regimes B/160/120/4 and B/170/60/4 producing the highest content (43.7–44.2%). The relative increase in glucan content was due to sample ML and acetone extractives being washed out of the wood structure. The content of arabinan was down from 0.7% for untreated wood to 0.1–0.3% for TM birch, but mannan content was 0.9–1.3%. Between all TM regimes, significant changes or correlations were not observed. Birch wood modified under the B/160/120/4 and B/170/60/4 regimes showed the greatest reduction in xylan content (15.5–16.2%). Both treatments resulted in identical acetyl group cleavage, with amounts as low as 2.4%. Also, regime B/160/90/4 resulted in substantial xylan destruction of 18.9%, whereas acetyl group cleavage occurred at the same level as in other treatments (3.2–4.2%). The degradation of hemicellulose is dependent on its composition; arabinoxylan, predominant in hardwoods, breaks down more quickly than galactoglucomannan, present in softwoods [32]. Compared to main-chain sugars like xylose, mannose, and glucose, side-chain sugars like arabinose and galactose are more labile [33]. Short rotation teak (SRT) and long rotation teak (LRT) hemicellulose content decreased from 26.3 to 10.7% for SRT and 27.6 to 9.6% for LRT after heat treatment at 220 °C for 20 h under nitrogen atmosphere, while the relative content of cellulose increased from 41.1 to 46.0% for SRT and 39.9 to 48.1% for LRT. For SRT and LRT, the relative lignin content grew from 32.6 to 43.3% and from 32.5 to 42.3%, respectively. After heat treatment, the total extractive contents of SRT and LRT fell by 3.8% and 30.4%, respectively. It was due to the evaporation of volatile compounds [8].

We believed that the breakdown of structural components would improve the hydrophobicity of TM wood. Our previous studies showed that regime B/170/60/4 had the best anti-swelling efficiency (ASE) (63% after the fifth cycle) compared to regimes B/160/120/4 and B/160/90/4, which had ASE of 45 and 33%, respectively [5]. Chemical composition changes did not allow for the prediction of wood water-related properties following TM in nitrogen. Practical testing of TM wood utilizing a variety of procedures is required to fully characterize the resulting material.

The TM of Scots pine wood also caused changes in chemical components (Table 4). Pine wood showed a lower ML (3.9–9.0%) after TM than did birch wood (5.9–12.0%). At 180 °C (regimes P/180/30/5 and P/180/60/5) and 170 °C with maximum T_max_ (regimes P/170/120/4 and P/170/120/6), the highest ML of pine wood (7.6–9.0%) was obtained. At 160 °C (regimes P/160/120/5 and P/160/180/5), the lowest ML was recorded. In our earlier research, TM pine wood showed greater loss in the tangential direction (2.9–4.5%) than in the radial direction (1.5–2.5%), with volumetric changes ranging from 4.4% to 6.9% [5]. The inorganic part (ashes) of untreated and all TM wood specimens was 0.2–0.3%; hence, the data are not given.

Scots pine TM in a nitrogen environment enhanced the quantity of acetone extractives, lignin, glucan, and mannan, but xylan and acetyl groups were reduced. The proportion of acetone-soluble extractives in TM wood increased to 1.8–4.7% as compared to untreated pine wood (0.4%). However, the total amount was considerably lower than that of TM birch wood. The maximum amount of extractives (4.7%) was recovered from wood TM in regime P/180/60/5, while the 30 min treatment also revealed a significant extractive content (3.3%). Although regimes P/170/120/4 and P/170/120/6 had relatively high ML, their extractive contents were only 2.9 and 2.7%, respectively. The lignin content increased after all treatments except P/160/120/5; however, the rise was not considerable and did not follow a clear trend depending on the TM parameters utilized. 

The relative amount of glucan following TM in nitrogen increased for all treatments, with the exception of regime P/170/60/4, which showed no significant increase. The regimes P/170/90/4, P/170/120/4, and P/170/120/6 produced the highest yields (45.8–46.6%). Mannan content increased throughout all treatments except P/170/120/4 and P/180/30/5. The xylan amount was lowered from 6.3% in untreated wood to 5.0–5.9% in TM wood. The regimes with the lowest xylan content were P/170/120/4 and P/180/60/5. Untreated pine wood has a significantly lower acetyl group concentration (1.6%) than birch wood (4.7%). Regimes P/170/120/4 and P/180/60/5 likewise had the lowest acetyl group level, 1.0–1.1%. The concentration of galactan and arabinan decreased from 1.5 and 1.4% in untreated pine wood to 0.9–1.3% and 0.1–0.5% in TM pine, respectively. Significant differences between TM regimes were not identified; hence, these data are not included. 

Our earlier research found that the ASE of regime P/180/60/5 was the greatest (51% after the fifth cycle), indicating the most significant structural alterations following TM. Regimes P/170/120/4, P/170/90/6, P/170/120/6, and P/180/30/5 had slightly lower ASE (46 to 48%) [5]. The association shows that TM pine samples with the highest ML and acetone extractives content and the lowest xylan and acetyl group content had the highest ASE values. As a result of the thermal degradation of chemical components, particularly xylan, pine wood’s water-related qualities improved.

Given that the concentration of xylan, arabinan, galactan, and acetyl groups in TM pine wood dropped by 3–4%, it is reasonable to conclude that ML is mostly a result of heat breakdown of natural pine resin. This is further supported by the comparatively low acetone-soluble extractives concentration in TM pine wood, which is analogous to the previously described compound reduction.

### 3.2. Mechanical Strength

The density of birch wood decreased as a result of TM (Table 5); this was caused by the degradation of wood’s structural components, primarily xylan and acetyl groups (Table 3). The density of air-dried, untreated silver birch wood was 652 ± 18 kg×m^−3^. Density was lowered across all TM samples. As compared to native wood for regimes B/160/120/4, B/170/30/4, and B/170/60/4, a significant drop was detected in the density of TM birch, which also had the highest ML after TM (Table 2), indicating a relationship between these parameters. The MOR of untreated birch was 124 MPa, which was lowered by 15–42% for all TM specimens. Regimes B/160/120/4, B/170/30/6, and B/170/60/4 produced the lowest MOR values (72–85 MPa). The decrease in MOR was primarily due to the breakdown of hemicelluloses during TM. Hemicelluloses are depolymerized into oligomers and monomers using hydrolysis processes. This involves the breakdown of the main-chain components, mannose, glucose, and xylose, after the cleavage of the side-chain elements, arabinose and galactose. Furfural and hydroxyl-methyl-furfural are produced by dehydrating the related pentoses and hexoses, respectively [20]. Our findings (Table 3) supported this, as they showed significant xylan degradation and acetyl group cleavage. Following TM under regimens B/150/120/5 and B/160/90/4, the MOR was reduced. TM at 150 °C generated small changes in birch wood’s chemical composition and had a negligible effect on MOR.

The untreated birch MOE was 13,300 MPa, and the TM process resulted in an increase in the average MOE values of birch wood based on the treatment parameters. Only regime B/160/60/4 showed an insignificant reduction. The highest MOE (14,700–15,100 MPa) was observed for specimens following TM under regimes B/150/120/5, B/160/90/4, and B/170/30/6. However, the MOE values had substantial error margins, and the differences between all TM regimes were insignificant.

The MOR of beech wood (with an initial density of 880 kg×m^−3^) dropped from 166 N×mm^−2^ to 110, 90, and 80 N×mm^−2^, respectively, after 1.5, 2.5, and 3.5 h of TM at 185 °C under a nitrogen flow at 10 bar pressure. After 2.5 h of TM at 185 °C in nitrogen, the MOR and MOE of beech wood (with an initial density of 700 kg×m^−3^) decreased from 110 to 70 N×mm^−2^ and 12,000 to 11,000 N×mm^−2^, respectively. After 2.5 h of TM at 195 °C, the MOR and MOE of poplar wood (with initial density 410 kg×m^−3^) decreased from 57 to 53 N×mm^−2^ and 7600 to 7500 N×mm^−2^, respectively. After 2.5 h of TM at 195 °C, the MOR of birch wood (with a lower initial density of 560 kg×m^−3^) decreased from 110 to 100, but the MOE increased from 13,000 to 14,000 N×mm^−2^ [6]. MOR decreased after TM in nitrogen, whereas MOE values varied. However, in our study, the TM of birch wood in nitrogen led to a greater reduction in MOR.

The tangential surface Brinell hardness of untreated and TM birch wood was substantially higher than that of the radial surface. TM birch wood’s tangential surface had a greater Brinell hardness than that of untreated wood, with the exception of regimes B/160/120/4 and B/170/60/4. It is difficult to explain why the Brinell hardness of the tangential surface improved after TM under regimes B/160/60/5, B/160/90/3, and B/160/90/4. SEM pictures indicated that silver birch (*Betula pendula*) wood morphological elements (libriform, vessels, rays, and yearly rings) had a significant decrease in size after 1 h of TM in saturated steam at 160 °C. The linear lumen sizes decreased more in the radial direction (2.9%) than in the tangential direction (0.5%). After being treated at 180 °C, the wood’s morphological structure started to disintegrate [34]. Perhaps the radial shrinkage of birch wood at 160 °C compacted the tangential surface and generated a modest rise in Brinell hardness. The microstructural alterations of birch wood caused by TM in saturated steam at 160 °C for 1 h were investigated using micro X-ray computed tomography. Above all studied wood species, birch displayed 16% ML and the largest volume loss (19%) after TM; however, porosity after TM was reduced from 34% to 29% [35]. As a result, the destruction of chemical components and microstructural changes following the TM process did not always result in a simultaneous drop in mechanical strength in all anatomical directions of the wood.

Radial surface Brinell hardness was comparable between untreated and most TM wood specimens, taking error limits into account, with the exception of regimes B/160/120/4, B/170/30/4, and B/170/60/4. These treatments yielded the lowest xylan and acetyl group amounts (Table 3). TM in nitrogen at T_max_ 160 °C and maximum time at T_max_ (120 min), as well as treatments at 170 °C (30 and 60 min), resulted in the most extensive thermal degradation of birch wood components. 

As a result, MOR and Brinell hardness were lowered significantly. The Brinell hardness of European beech (*Fagus sylvatica* L.) wood was lowered from 44.8 to 39.9 N×mm^−2^ after 6 h of TM in a nitrogen environment at 190 °C. However, the specific anatomical surface evaluated for indentation was not mentioned [21]. Beech wood density (681 ± 20 kg×m^−3^) utilized in that investigation was equivalent to that of silver birch wood; however, it recorded more than 2 fold higher Brinell hardness than birch wood. The anatomical structure of beech wood is comparable to that of birch wood; hence, it is unclear why such disparate results were achieved.

The density of pine wood after TM decreased (Table 6). It was a consequence of the ML caused by the degradation and evaporation of natural resins and destruction of the structural components of wood, mainly xylan and acetyl groups (Table 4). The density of air-dried untreated Scots pine wood was 581 ± 22 kg×m^−3^, which decreased following all TM treatments. However, the variations were not statistically significant. There was no correlation between ML and density for TM pine wood, as was the case with birch wood.

The MOR of untreated Scots pine was 98 MPa, which was lowered by 2–32% among all TM specimens. The MOR values for P/170/120/4, P/170/90/6, P/170/120/6, P/180/30/5, and P/180/60/5 were significantly lower (67–79 MPa) than those for untreated pine wood. The average MOR values following TM at 170 °C with both initial pressures (4 and 6 bar) tended to decrease as the time at T_max_ increased. However, the changes between these regimes were insignificant.

The MOE of untreated Scots pine was 12,400 MPa, and MOE values decreased when the TM process was applied. Only the regimes P/160/180/5 and P/170/60/4 showed insignificant improvement. Specimens following TM at 170 °C and time at T_max_ 120 min with both beginning pressures (4 and 6 bar) had the lowest MOE (10,000–11,200 MPa). However, all MOE values were in a similar range, and the variations were not significant.

Pine wood’s MOR and MOE decreased from 110 to 80 N×mm^−2^ and 16,000 to 13,000 N×mm^−2^ after 3 h of TM at 185 °C in nitrogen [6]. Scots pine’s MOR dropped from 88.7 to 85.9 N×mm^−2^ following TM at 165–185 °C and T_max_ 0–90 min, whereas its MOE increased from 9660 to 10,660 N×mm^−2^. Radiata pine and Norway spruce exhibited similar tendencies in terms of MOR and MOE after TM [20]. Overall, our findings are similar to those of previously mentioned studies, and TM in nitrogen in a closed pressured process causes loss of MOR for Scots pine wood. However, prior investigations have found inconsistent MOE changes following TM. Still, it should be noted that the error limitations in these tests were wide, and the rise in MOE following TM was not significant.

The Brinell hardness of the radial surface of untreated and TM pine wood was much higher than that of the tangential surface, and the pattern was opposite when compared to silver birch. The Brinell hardness of the tangential surface of Scots pine was 13.8 N×mm^−2^ before treatment. For the regimes P/170/60/4, P/170/90/4, P/170/120/4, and P/170/120/6, it was slightly lower after TM. The Brinell harness was altered by the rest of the treatments, although not significantly. TM Scots pine wood’s radial surface Brinell hardness increased, particularly under regimes P/170/90/4, P/170/120/4, P/170/60/6, and P/170/90/6. 

Using micro X-ray computed tomography, the microstructure of pine wood was investigated after TM in saturated steam at 160 °C for 1 h. Scots pine wood demonstrated 17% volume loss and 15% ML, but the porosity (28%) following TM remained constant [35]. In our previous research employing TM of pine wood, the tangential direction (2.9–4.5%) showed a higher reduction than the radial direction (1.5–2.5%) [5]. It is likely that greater tangential direction shrinkage after TM leads to microstructure rearrangement in a denser structure that raises the radial surface’s hardness.

The Brinell hardness of Scots pine (*Pinus sylvestris* L.) decreased from 40.7 to 37 N×mm^−2^ after 6 h of TM in nitrogen atmosphere at 190 °C, but the changes were not statistically significant. The anatomical surface that was evaluated for indentation was not mentioned; however, the density (595 ± 7 kg×m^−3^) was quite similar to that of the pine wood we utilized for our research [21]. Since the approach employed by the authors of that study was the same, it is impossible to explain how they arrived at such values. It is confusing since they quoted a different study in which the Brinell hardness of pine wood (heartwood and sapwood) was measured following a saturated steam treatment. The hardness of sapwood increased from 11.6 MPa to 12.0 MPa, whereas that of heartwood decreased from 11.4 MPa to 10.7 MPa after 3 h of TM at 150 °C. Increasing the TM temperature to 180 °C decreased wood hardness to approximately 9 MPa in both tested zones [36]. These values are comparable to our findings. However, after TM in nitrogen under pressure, pine wood Brinell hardness remained constant across the tangential surface while increasing significantly for the radial surface. The Brinell hardness parallel to the grain of Scots pine after TM at 165–185 °C and time at T_max_ 0–90 min was obviously increased from 36 to 53.2 N, whereas the hardness perpendicular to the grain increased slightly from 17.5 to 18.4 N after TM [20]. In that investigation, the difference between grain directions was doubled for untreated wood and tripled after TM. The reasons for the variations in grain orientations and Brinell hardness improvements following TM were not fully explained.

## 4. Discussion

When raw solid wood samples are selected within a specified density range, the mechanical strength falls within a specific range of values. When the selected boards are thermally modified and their mechanical strength evaluated, the error limitations grow much broader, and the average values can be misleading. Obviously, the chemical transformation of wood components in the TM process varies not only between treatment regimes. Furthermore, the chemical microstructure of each particular board following TM in nitrogen can vary. This is also demonstrated by the dispersion of ML within a single TM treatment regime, and the average value does not allow for general assumptions regarding the treatment. Specifically, a second investigation might be undertaken to determine how the mechanical strength and chemical composition of wood boards change following TM in nitrogen, depending on their position in the modification chamber. A large study would be appropriate for only a few treatment regimes. This might be investigated for the regimes that had the best water-related characteristics in our previous study [5]. These specimens revealed the most substantial chemical structure changes in this study, enabling considerably easier detection of variations between different boards after TM. This, as well as previous research, demonstrated that using this study technique, it is impossible to forecast wood water-related properties after TM in nitrogen based on chemical composition changes. Practical testing of TM wood utilizing a variety of procedures is required to fully characterize the resulting material.

The Brinell hardness of silver birch and Scots pine showed an opposing trend after nitrogen TM treatment. Obviously, the microstructure of untreated birch wood differs from that of pine wood, as do the alterations after TM. To support or reject this hypothesis, future study should include SEM images of both wood species after TM under various treatment regimes. However, for solid wood, it is a time-consuming experiment that should be well prepared before beginning in order to collect valuable information for comparison.

## 5. Conclusions

TM of silver birch and Scots pine wood produced ML due to thermal degradation of wood chemical components. The most significant changes in birch wood after TM were observed in the content of acetone extractives, xylan, and acetyl groups, while glucan and lignin showed less significant changes. Scots pine TM in nitrogen environment enhanced the quantity of acetone extractives, lignin, glucan, and mannan, but xylan and acetyl groups were reduced. The most substantial changes in the chemical structure of silver birch wood were detected after TM for the maximum time at 160 and 170 °C (regimes B/160/120/4 and B/170/60/4). If all other process parameters remained constant, increasing the initial pressure resulted in a drop in xylan and acetyl group content while increasing ML. As a result, the chemical composition of birch wood after TM was influenced by all process parameters. The most substantial changes in the chemical structure of Scots pine wood occurred following treatments at 170 °C and 180 °C for the maximum period at T_max_ (regimes P/170/120/4, P/170/120/6, and P/180/60/5). The chemical composition of pine wood was not significantly affected by the initial pressure rise (4 to 6 bar) at 170 °C.

The MOR of both wood species was reduced after TM in nitrogen, while the MOE changes were minor. Scots pine TM resulted in a smaller total MOR loss (2–32%) than TM of birch wood (15–42%). The Brinell hardness of TM birch wood’s tangential surface was significantly higher than that of the radial surface, although Scots pine wood showed the opposite pattern. The reason for this is that the microstructural rearrangements after TM in nitrogen differ between both examined wood species.

Overall, softwood (Scots pine) was more thermally stable than hardwood (silver birch), as evidenced by lower ML, extractive content, and the loss of xylan and acetyl groups. TM birch and pine wood specimens with the highest ML, acetone soluble extractives concentration, and the lowest xylan and acetyl group content had the lowest MOR and Brinell hardness values. We expected pentosans (primarily xylan) to be the primary component degraded after TM of birch wood, while pine wood would be subjected to more thermal destruction of hexosans (glucan and mannan). Although it was established for birch wood, the chemical composition of pine wood after TM revealed no substantial loss of glucan or mannan content. The quantity of xylan, arabinan, galactan, and acetyl groups was reduced by 3–4% in TM pine wood, whereas ML after TM was substantially greater (3.9–9.0%) and was mostly generated by the thermal degradation and evaporation of natural pine wood resins.

## Figures and Tables

**Table 1:** Thermal modification parameters for silver birch wood [5].

Treatment Regime	T_max_ (°C)	Time at T_max_ (min)	Initial Pressure (bar)	Max Pressure (bar)
B/150/120/5	150	120	5	10.2
B/160/60/4	160	60	4	11.8
B/160/60/5	160	60	5	14.2
B/160/90/3	160	90	3	10.0
B/160/90/4	160	90	4	12.0
B/160/120/4	160	120	4	12.6
B/170/30/3	170	30	3	12.7
B/170/30/4	170	30	4	13.0
B/170/30/6	170	30	6	16.9
B/170/60/4	170	60	4	13.2

**Table 2:** Thermal modification parameters for Scots pine wood [5].

Treatment Regime	T_max_ (°C)	Time at T_max_ (min)	Initial Pressure (bar)	Max Pressure (bar)
P/160/120/5	160	120	5	12.7
P/160/180/5	160	180	5	13.2
P/170/60/4	170	60	4	12.5
P/170/90/4	170	90	4	12.7
P/170/120/4	170	120	4	12.9
P/170/60/6	170	60	6	15.4
P/170/90/6	170	90	6	15.8
P/170/120/6	170	120	6	16.2
P/180/30/5	180	30	5	16.4
P/180/60/5	180	60	5	16.8

**Table 3:** Chemical composition of TM silver birch wood.

Treatment Regime	Mass Loss (%)	Chemical Components (%)				
		Acetone Extractives	Lignin	Glucan	Xylan	Acetyl Groups
Untreated	–	1.9 ± 0.1	23.4 ± 0.2	39.3 ± 0.1	21.4 ± 0.1	4.7 ± 0.0
B/150/120/5	7.0 ± 2.3	4.5 ± 0.2	23.5 ± 0.1	39.9 ± 0.3	20.8 ± 0.1	3.9 ± 0.0
B/160/60/4	7.7 ± 2.7	4.0 ± 0.2	23.8 ± 0.1	41.2 ± 0.3	21.0 ± 0.3	4.2 ± 0.1
B/160/60/5	9.5 ± 3.1	6.7 ± 0.2	21.6 ± 0.2	41.4 ± 0.1	20.9 ± 0.1	3.4 ± 0.0
B/160/90/3	5.9 ± 2.2	6.5 ± 0.1	22.0 ± 0.1	41.5 ± 0.1	20.5 ± 0.1	3.5 ± 0.0
B/160/90/4	7.3 ± 2.9	6.5 ± 0.2	22.8 ± 0.1	41.5 ± 0.1	18.9 ± 0.1	3.4 ± 0.0
B/160/120/4	10.1 ± 1.7	12.6 ± 0.3	20.1 ± 0.1	44.2 ± 0.1	16.2 ± 0.1	2.4 ± 0.0
B/170/30/3	8.6 ± 2.6	7.9 ± 0.1	22.7 ± 0.1	40.7 ± 0.1	20.5 ± 0.1	3.2 ± 0.0
B/170/30/4	9.6 ± 2.8	6.1 ± 0.2	24.2 ± 0.1	42.5 ± 0.1	19.3 ± 0.1	3.2 ± 0.0
B/170/30/6	9.3 ± 2.9	8.3 ± 0.2	23.1 ± 0.1	41.6 ± 0.1	20.4 ± 0.1	3.3 ± 0.1
B/170/60/4	12.0 ± 2.2	9.6 ± 0.2	24.0 ± 0.1	43.7 ± 0.4	15.5 ± 0.3	2.4 ± 0.1

**Table 4:** Chemical composition of TM Scots pine wood.

Treatment Regime	Mass Loss (%)	Chemical Components (%)					
		Acetone Extractives	Lignin	Glucan	Xylan	Mannan	Acetyl Groups
Untreated	–	0.4 ± 0.1	27.6 ± 0.1	42.9 ± 0.2	6.3 ± 0.2	10.3 ± 0.1	1.6 ± 0.0
P/160/120/5	3.9 ± 0.9	1.8 ± 0.2	27.0 ± 0.7	45.6 ± 0.3	5.9 ± 0.1	11.7 ± 0.0	1.5 ± 0.0
P/160/180/5	4.7 ± 1.3	2.2 ± 0.2	28.9 ± 0.1	45.9 ± 0.1	5.3 ± 0.1	11.3 ± 0.1	1.3 ± 0.0
P/170/60/4	4.9 ± 1.0	2.4 ± 0.1	30.2 ± 0.1	43.1 ± 0.2	5.4 ± 0.1	12.6 ± 0.1	1.2 ± 0.0
P/170/90/4	6.6 ± 1.5	2.5 ± 0.2	29.1 ± 0.2	46.6 ± 0.2	5.4 ± 0.0	11.0 ± 0.1	1.3 ± 0.0
P/170/120/4	7.9 ± 1.3	2.9 ± 0.2	31.2 ± 0.2	45.9 ± 0.3	5.1 ± 0.1	10.1 ± 0.1	1.1 ± 0.0
P/170/60/6	6.3 ± 1.2	2.7 ± 0.1	29.1 ± 0.1	43.7 ± 0.0	5.7 ± 0.1	13.3 ± 0.1	1.3 ± 0.0
P/170/90/6	6.0 ± 1.3	2.8 ± 0.2	29.4 ± 0.1	44.5 ± 0.1	5.2 ± 0.0	11.0 ± 0.1	1.3 ± 0.0
P/170/120/6	7.8 ± 1.2	2.7 ± 0.1	29.5 ± 0.1	45.8 ± 0.2	5.5 ± 0.1	11.0 ± 0.0	1.3 ± 0.0
P/180/30/5	7.6 ± 1.6	3.3 ± 0.2	32.9 ± 0.1	44.6 ± 0.1	5.5 ± 0.1	10.2 ± 0.1	1.1 ± 0.0
P/180/60/5	9.0 ± 1.6	4.7 ± 0.2	31.4 ± 0.1	44.5 ± 0.2	5.0 ± 0.1	11.7 ± 0.2	1.0 ± 0.0

**Table 5:** Mechanical strength of TM silver birch wood.

Treatment Regime	Density (kg×m^−3^)	MOR (MPa)	MOE (MPa)	Brinell Hardness (N×mm^−2^)	
				Tangential Surface	Radial Surface
Untreated	652 ± 18	124 ± 8	13,300 ± 1100	21.5 ± 1.0	19.1 ± 1.0
B/150/120/5	614 ± 49	105 ± 24	14,700 ± 1500	21.8 ± 1.9	17.4 ± 3.2
B/160/60/4	611 ± 32	86 ± 15	12,500 ± 1100	23.5 ± 2.3	18.4 ± 2.6
B/160/60/5	622 ± 22	100 ± 25	13,800 ± 1400	24.5 ± 2.0	20.4 ± 2.0
B/160/90/3	642 ± 33	93 ± 19	14,400 ± 1400	27.3 ± 2.9	20.7 ± 2.4
B/160/90/4	622 ± 16	110 ± 27	14,900 ± 1000	25.4 ± 2.2	18.9 ± 2.3
B/160/120/4	576 ± 47	72 ± 11	13,600 ± 1200	17.9 ± 2.4	15.4 ± 2.0
B/170/30/3	601 ± 16	98 ± 16	14,400 ± 700	22.6 ± 1.2	17.0 ± 1.4
B/170/30/4	581 ± 27	90 ± 20	13,700 ± 1100	22.0 ± 2.5	14.7 ± 2.6
B/170/30/6	632 ± 44	83 ± 20	15,100 ± 1200	24.9 ± 3.6	19.6 ± 2.6
B/170/60/4	509 ± 22	85 ± 24	14,400 ± 1800	17.0 ± 2.6	13.6 ± 2.6

**Table 6:** Mechanical strength of TM Scots pine wood.

Treatment Regime	Density (kg×m^−3^)	MOR (MPa)	MOE (MPa)	Brinell Hardness (N×mm^−2^)	
				Tangential Surface	Radial Surface
Untreated	581 ± 22	98 ± 5	12,400 ± 1000	13.8 ± 1.2	14.3 ± 0.9
P/160/120/5	578 ± 17	85 ± 16	11,800 ± 900	16.5 ± 2.0	18.9 ± 1.6
P/160/180/5	570 ± 43	93 ± 15	12,700 ± 1300	17.0 ± 2.7	18.7 ± 4.0
P/170/60/4	549 ± 44	96 ± 18	13,300 ± 1100	13.5 ± 1.7	16.4 ± 2.6
P/170/90/4	577 ± 19	83 ± 18	12,100 ± 700	13.5 ± 1.7	21.3 ± 3.7
P/170/120/4	560 ± 12	70 ± 18	11,200 ± 1100	13.5 ± 1.6	20.9 ± 3.1
P/170/60/6	581 ± 10	88 ± 18	11,700 ± 800	14.6 ± 1.2	20.8 ± 2.0
P/170/90/6	547 ± 25	78 ± 18	11,200 ± 900	14.4 ± 3.2	20.3 ± 3.5
P/170/120/6	541 ± 19	67 ± 19	10,000 ± 700	13.1 ± 0.9	18.4 ± 2.1
P/180/30/5	555 ± 35	79 ± 14	12,400 ± 1100	16.4 ± 1.7	18.6 ± 2.2
P/180/60/5	564 ± 52	75 ± 21	11,800 ± 1800	14.0 ± 4.4	17.5 ± 4.9

## Data Availability

The raw data supporting the conclusions of this article will be made available by the authors on request.
